# Supplementary material for: PROTEIN-PROTEIN INTERACTION NETWORKS CAN BE HIGHLY SENSITIVE TO THE MEMBRANE PHASE TRANSITION
Source: bioRxiv. 2025 Oct 31:2025.10.30.685611. Preprint. [Version 1] doi: 10.1101/2025.10.30.685611 (PMC12636333; doi:10.1101/2025.10.30.685611)
Supplement: Supplement 1 [file NIHPP2025.10.30.685611v1-supplement-1.pdf]

bioRxiv preprint doi: https://doi.org/10.1101/2025.10.30.685611; this version posted October 31, 2025. The copyright holder for this preprint (which was not certified by peer review) is the author/funder, who has granted bioRxiv a license to display the preprint in perpetuity. It is made available under aCC-BY 4.0 International license.

Supplementary Information

A Simulation Framework: MemTropy

Here, we summarize the structure and function of the simulation framework used for the calculations carried out in this manuscript. The simulation framework in its entirety with more detailed instructions on its use is available at `https://gitlab.com/taylor-schaffner/memtropic`. The framework consists of an engine written in C++ wrapped into Cython where it is configured and called. In it's current form, it may be imported to an interactive python session or executed as script. The full suite contains a user-friendly GUI with an on-going implementation of tools for exploring interaction networks among extended objects in a 2D membrane driven by entropy.

B Data and Analysis Files

All post-simulation data analysis was carried out using MATLAB. Analysis code and data files can be found organized by figure at `https://gitlab.com/taylor-schaffner/ppi-paper-figure-data`.

C Detailed Balance on the Membrane

Equilibration of the 2D Ising model membrane in our model is done in a manner that conserves the average spin value (magnetization) of the system. In other words, rather than flipping spins, spins on the lattice are exchanged. In each case, the detailed balance condition is satisfied by using the standard Metropolis acceptance probability

$$P(A \rightarrow B) = \text{Min}[1, \exp(-\beta(E_B - E_A))] \tag{3}$$

Moves that do not increase energy are accepted, and moves that do are accepted according to a Boltzmann distribution. In each implementation case considered in the following, the same acceptance probability is used with only changes in the definitions of state A and B.

C.1 Local Kawasaki Algorithm

Individual spins, here labeled lipids, migrate in our model by swapping with their neighbors according to 3. For a given move, the states A and B differ only by the 6 modified bonds following the proposed exchange. In this way, lipids migrate in a way that conceptually replicates diffusion in the membrane.

C.2 Non-local Kawasaki Algorithm

Prior to the primary run-time in which protein-protein interaction network data is collected for a system, time to thermal equilibrium steady-state may be decreased considerably by first performing non-local spin swaps of the individual lipids. These moves similarly obey 3 but in this case, the difference between states A and B consists of 8 modified bonds.

C.3 Diffusion of Membrane Inclusions

Following an algorithm previously utilized for studies on lattice-based colloidal chemistry simulations [26], we model diffusion of a membrane inclusion as a non-local, cluster spin exchange. After selecting a random inclusion (PPI component), one of four cardinal directions is randomly chosen, and an attempt is made to simultaneously swap the membrane spins adjacent to the inclusion's leading edge (outside the inclusion) with the corresponding spins on the back edge on the inclusion (inside inclusion). The difference between states A and B in this case is again the set of bonds altered–the number of which increases with disk radius.

C.4 Thermal equilibration

C.4.1 Simulations with fixed order parameter

In the majority of the results presented, the magnetization order parameter/fraction of dark spins is fixed for the runtime. In these systems, we ensure that the system is in thermal equilibrium before data collection. One means by which we achieve this is to equilibrate the membrane sans disks via non-local Kawasaki swaps, and then add the disks onto the lattice while minimizing order-parameter changes–i.e. by placing a dark phase-preferring disk into a pool of dark spins only.

bioRxiv preprint doi: https://doi.org/10.1101/2025.10.30.685611; this version posted October 31, 2025. The copyright holder for this preprint (which was not certified by peer review) is the author/funder, who has granted bioRxiv a license to display the preprint in perpetuity. It is made available under aCC-BY 4.0 International license.

C.4.2 Simulations with disks changing partitioning

In systems with an interaction network that modifies the target's partition preference, we can thermally equilibrate before hand, but the partition change will always break the equilibrium. Furthermore, while the observed phenomenon we present here and call 'pocketing' will occur from simulations initiated at thermal equilibrium, we note that its occurrence may be greatly increased by initializing the system with conducive thermal inhomogeneities. The results in the domain-changing section were initialized by first placing the disks on the lattice in a uniformly distributed manner and then performing non-local Kawasaki sweeps to attempt to equilibrate the surrounding solvent. The result is a phase-separated but segmented lattice that is highly conducive to forming pockets as many recruiters become isolated.

D Cross-Correlation Functions

All correlation functions considered in the results presented are two-dimensional (cross) correlation functions computed as described below. The computational outline considers a general set of two signal matrices A and B, and then the remaining sections define what those matrices contain in each case considered.

D.1 Computation of 2D Cross-Correlations

We begin with two signal matrices A and B and assume that they are square and share the same dimensions, $N \times N$. We make use of discrete Fourier transform (DFT) techniques to efficiently compute two-dimensional cross-correlation functions.

1. zero-padding
   All data considered in this manuscript originate from systems with periodic boundary conditions. As a result, we may forgo zero-padding signal matrices before computing the correlations.
2. zero-mean signal
   Now we define a set of shifted signal matrices such that each has a mean of zero.
   $$A = A - \langle A \rangle$$
   $$B = B - \langle B \rangle$$
   With these definitions, we are ready to proceed with the correlation calculation.
3. Fourier transform each signal
   Discrete Fourier transforms of $A = A(x, y)$ and $B = B(x, y)$ are computed to obtain the frequency space representations $A_k(p, q)$ and $B_k(p, q)$
   $$A_k(p,q) = \sum_{x=0}^{N-1} \sum_{y=0}^{N-1} A(x,y) e^{-\frac{\pi i}{N}(px+qy)}$$
   and similarly for $B_k(p, q)$.
4. compute the power spectrum
   The power spectrum denoted $G_k(p, q)$ is then computed by element-wise multiplication between $A_k(p, q)$ and the complex conjugate of $B_k(p, q)$.
   $$G_k(p,q) = A_k(p,q) B_k^*(p,q)$$
5. un-normalized cross-correlations
   The power-spectrum is then inverse-Fourier transformed to obtain the un-normalized two dimensional cross-correlation function in position space $\tilde{G}(x, y)$
   $$\tilde{G}(x,y) = \frac{1}{N^2} \sum_{p=0}^{N-1} \sum_{q=0}^{N-1} G_k(p,q) e^{\frac{\pi i}{N}(px+qy)}$$
6. normalization
   To normalize the two dimensional cross-correlation function, we divide the result by the square root of the product of the squared-sum of each signal matrix, i.e. each density.
   $$G(x,y) = \frac{\tilde{G}(x,y)}{\sqrt{\sum_{x,y} A(x,y)^2 \sum_{x,y} B(x,y)^2}}$$

bioRxiv preprint doi: https://doi.org/10.1101/2025.10.30.685611; this version posted October 31, 2025. The copyright holder for this preprint (which was not certified by peer review) is the author/funder, who has granted bioRxiv a license to display the preprint in perpetuity. It is made available under aCC-BY 4.0 International license.

D.2 Computation of Radial Average

To more easily digest the information contained in the computed two-dimensional cross-correlation functions, we reduce the dimensionality by radially averaging.

1. shift zero-frequency to the center
   Before radially-averaging $G(x, y)$, we perform what is commonly referred to as a Fourier shift which involves rotating the data such that the interesting components are centered. For our two-dimensional, data this is simply a swap of the first quadrant with the third quadrant and a swap of the second quadrant with the fourth.
2. radially average
   The shifted $G(x, y)$ is radially-averaged where the radius r is defined as the distance from the center of the cross-correlation matrix. Note that our lattice (and hence signal matrices) is even dimensions and, in the discrete Fourier transform algorithms we use, $(0, 0)$ is defined to be the spatial center. Thus following the Fourier shift, the new center is located at index $(N/2, N/2)$, setting $r = 0$ for the radial average. This gives us $G(r)$.

D.3 Protein-Protein Cross-Correlations

In the case protein-protein cross-correlation calculations we consider in the main results the cross-correlation between the target component and its activator and between the target component and its inactivator. For instance, if we consider the activator-target cross-correlation specifically, matrix A is then a matrix corresponding to the standard simulation lattice but with 1's at all sites containing an activating component and 0's elsewhere and matrix B is a similar matrix with 1's and 0's denoting the location of all targets.

D.4 Spin-Spin Correlations

Spin-spin correlation functions are computed using the same procedure outlined above, but in such a calculation, $A = B$. Additionally, in this signal matrix, up spins are represented by 1 while down spins are represented by -1.

E Varying Component Size

E.1 Fitting activity as a function of temperature for various PPI component sizes

When comparing the activity response to changes in temperature for various PPI component sizes, we fit equation 2 and obtained the following tabled fit values alongside the 95% confident interval bounds for each value. All fits were performed in MATLAB.

Fit Parameter	$r = 0$	$r = 1$	$r = 2$	$r = 3$
L	0.292 (0.273,0.311)	0.184 (0.168,0.200)	0.202 (0.185,0.220)	0.138 (0.124,0.152)
R	0.565 (0.551,0.579)	0.737 (0.707,0.768)	0.793 (0.770,0.816)	0.760 (0.741,0.778)
τ_0^{fit}	0.996 (0.993,0.999)	1.014 (1.011,1.016)	1.008 (1.007,1.010)	1.005 (1.003,1.006)
$\Delta\tau$	0.0209 (0.0177,0.0257)	0.0207 (0.0184,0.0236)	0.0165 (0.0147,0.0188)	0.0150 (0.0137,0.0166)

The coupling of inclusions (any disk with $r > 0$ in this context) to the 2D Ising membrane has the effect of making the system effectively *colder* than without them. This perturbation in our system is viewed as an increase in the true critical temperature of the system T_c to simplify the interpretation of τ throughout the manuscript. In the process of fitting the activity data in Fig. 3**A**, an estimate of this increased critical temperature τ_0^{fit} is calculated. This shift accounts for the x-axis shift in the activity curves. In practice, the approximate new critical point is fairly close to the original critical point provided that the PPI components occupy a reasonably small fraction (less than 2% for all cases considered here) of the total lattice. The exact numerical value of this shift is largely irrelevant to the qualitative arguments put forth in this work, so this discussion and the fit values are included primarily as a visual aid.

E.2 Maximum slope of activity vs temperature data

bioRxiv preprint doi: https://doi.org/10.1101/2025.10.30.685611; this version posted October 31, 2025. The copyright holder for this preprint (which was not certified by peer review) is the author/funder, who has granted bioRxiv a license to display the preprint in perpetuity. It is made available under aCC-BY 4.0 International license.

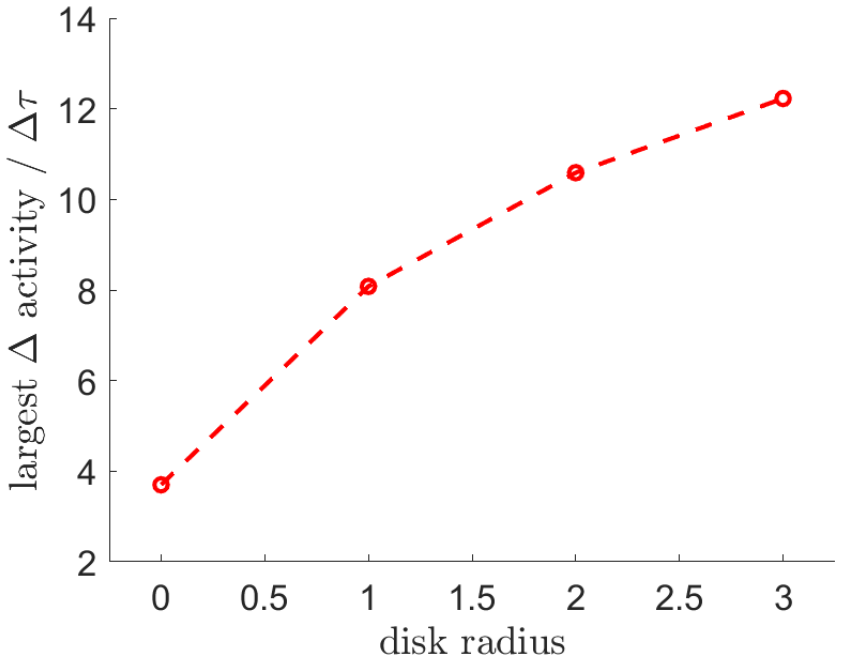

Figure 6: An alternative view the of activity's sensitivity gain as a function of component radius can be observed by looking at the maximum slope of the activity vs temperature for each radii.
